# Potential roles of MNREAD acuity charts and contrast/glare sensitivity in Ranibizumab treatment of branch retinal vein occlusion

**DOI:** 10.1371/journal.pone.0235897

**Published:** 2020-07-10

**Authors:** Hirotaka Tanabe, Akira Obana, Sachiko Yamamoto, Kiyomi Ichikumi, Yuko Gohto, Takahiko Seto, Takanobu Moriyama

**Affiliations:** Department of Ophthalmology, Seirei Hamamatsu General Hospital, Shizuoka, Japan; Icahn School of Medicine at Mount Sinai, UNITED STATES

## Abstract

**Background/Objectives:**

To investigate the potential utility of MNREAD acuity charts and contrast/glare sensitivity (CGS) assessment for evaluating the efficacy of an initial treatment with ranibizumab (Lucentis®) for branch retinal vein occlusion (BRVO).

**Methods:**

Intravitreal injections of ranibizumab were administered in 43 eyes of 43 treatment-naïve patients with BRVO. Efficacy was assessed 1 month later. Best-corrected far/near visual acuity (BCFVA/BCNVA), MNREAD parameters (reading acuity [RA], maximum reading speed [MRS], critical print size [CPS]), CGS (CS/GS), and central macular thickness (CMT) in optical coherence tomography (OCT) before and after treatment were evaluated. The area (superior/inferior) affected by BRVO was determined by fluorescein angiography.

**Results:**

All parameters improved significantly following treatment (*p* < 0.05), and all MNREAD and CGS parameters were significantly correlated with BCVA in the treated eye before and after treatment (*p* < 0.01). The changes in BCFVA, BCNVA, MRS, and CS were significantly correlated with the amount of change in CMT (*p* < 0.007; r = 0.415, 0.528, -0.465, and -0.508, respectively). MRS exhibited a percentage change that was significantly correlated with that in CMT (*p* < 0.007; r = -0.511). Additionally, MRS exhibited the lowest threshold CMT (397 μm) at which the most significant change in improvement was observed. CMT was less likely to improve if BRVO occurred at a superior site than if it occurred at an inferior site (0.05 < *p* = 0.07 < 0.1).

**Conclusions:**

MNREAD and CGS testing were useful for evaluating BRVO treatment efficacy. MRS might be a valuable index for evaluating treatment success and making treatment decisions.

## Introduction

Intravitreous injection of an anti-vascular endothelial growth factor (VEGF) agent is the current standard treatment for macular edema secondary to branch retinal vein occlusion (BRVO) [1–5]. There are several regimens for anti-VEGF treatment, including fixed injection, injection as needed (*pro re nata*; PRN), and treat and extend injection regimens. The first clinical study of ranibizumab used a fixed monthly injection regimen [1]. However, in clinical practice the PRN regimen is widely used. In the PRN regimen, the change in macular edema evaluated by changes in retinal thickness and morphology by optical coherence tomography (OCT) and the change in visual acuity are the general criteria for determining treatment. Another important criterion is the degree of patient satisfaction. Physicians make treatment decisions based on not only visual acuity and OCT findings but also patient intention. Patient satisfaction is not always consistent with the changes in OCT findings and visual acuity. One reason for this discrepancy is that visual acuity is only one of several parameters related to visual function and does not fully represent the patient’s problem. Another reason is that the condition of the fellow eye, which compensates for the involved eye, can influence the patient’s need for treatment. Reading ability and contrast/glare sensitivity (CGS) are considered strongly related to patient satisfaction with treatment and thus affect treatment choice. Therefore, the relationships among these and other parameters in naive BRVO patients who receive ranibizumab as an initial anti-VEGF treatment warrant investigation.

Measures of visual function other than visual acuity after anti-VEGF treatment of BRVO have been reported in a few papers [6–9]. Suner et al. [6] reported improvements in reading speed as estimated by the reading speed test developed by the Macular Photocoagulation Study Group. Varma et al. [7] measured the NEI VFQ-25, another indicator of visual function, during BRVO treatment via intravitreal injection of ranibizumab. Preti et al. [8] reported that a single intravitreal bevacizumab injection improved contrast sensitivity (CS) in macular edema from BRVO. Pece et al. [9] reported that CS, MNREAD time, and reading fluency improved significantly in eyes treated with ranibizumab.

MNREAD was developed by the Minnesota Laboratory for Low-Vision Research, University of Minnesota, for low-vision patients who cannot be fully evaluated by visual acuity alone [10,11]. In patients with low vision, visual performance is often more useful than visual acuity for evaluating visual function. This evaluation is composed of three parameters: reading acuity (RA), maximum reading speed (MRS), and critical print size (CPS). MNREAD has been gradually recognized as among the most credible examinations in the ophthalmological field [12–14], and it has been used in many clinical studies of various diseases worldwide [15–19]. MNREAD has been translated into numerous languages [20–22]. The Japanese version of MNREAD, MNREAD-J, was collaboratively developed and established by the Department of Communication of Tokyo Woman’s Christian University in Japan and the Minnesota Laboratory for Low-Vision Research, University of Minnesota, USA [23].

In the present study, we investigated the potential utility of MNREAD acuity charts and CGS assessment for evaluating the efficacy of a single treatment with ranibizumab in treating BRVO patients. Furthermore, we explored differences in the improvements in visual parameters posttreatment based on the affected region (inferior/superior) of the fundus.

## Materials and methods

### Study design and target population

Forty-three eyes of 43 patients (22 males and 21 females aged 51–82 years [69.3 ± 8.3 years]) diagnosed with center-involving BRVO at Seirei Hamamatsu General Hospital were enrolled in this prospective, observational clinical study from April 2014 to September 2015. All patients had no history of any treatment for BRVO, including anti-VEGF therapy and laser photocoagulation. After the patients provided written informed consent, they were treated with an intravitreal injection of 0.5 mg of ranibizumab (Lucentis®, Alcon Pharma, Co., Ltd., Tokyo, Japan).

Each subject underwent a visual acuity test, reading ability test, and CGS test before treatment and one month after treatment. Best-corrected far or near visual acuity (BCFVA or BCNVA) was measured using a decimal visual acuity chart and converted to logMAR values for statistical analysis. Reading ability was evaluated using MNREAD-J [23]. The three parameters of MNREAD, i.e., RA, MRS, and CPS, were analyzed. CGS was measured by the CGT-2000 contrast/glare tester (Takagi Seiko, Co., Ltd., Nagano, Japan), and the area under the log CS function (AULCSF) was used to evaluate CS in a normal setting and in the presence of glare light. Central macular thickness (CMT) was measured by OCT (CIRRUS HD-OCT [Carl Zeiss Meditec, Inc., Tokyo, Japan]) before and after treatment. Fluorescein angiographic images were obtained to determine the precise location and area of BRVO before treatment. The fundus images of the fellow eyes revealed 1 case of a macular hole, 1 case of diabetic retinopathy, 2 cases of epiretinal membrane, 1 case of old BRVO, and 1 case of macular coloboma.

We adhered to the tenets of the Declaration of Helsinki, and the ethics committee of Seirei Hamamatsu General Hospital approved the protocol of this prospective, observational clinical study (registration no. 1563).

### Data analyses

The following five steps were performed using R version 3.0.3 [24].

1. Evaluation of the improvements in visual function parameters and CMT after treatment
  BCVA (BCFVA and BCNVA), MNREAD values (RA, MRS, and CPS), CGS (CS/GS), and CMT in the treated eyes were compared between before treatment and after treatment using paired *t*-tests and Wilcoxon signed-rank tests. In addition, binocular MNREAD values were compared between before and after treatment.
2. Identification of the relationships between BCVA and other visual function parameters in treated eyes
  Correlations between BCVA (BCFVA and BCNVA) and other visual function parameters (RA, MRS, CPS, CS, and GS) before and after treatment were determined and analyzed using Pearson’s correlation coefficient analysis.
3. Identification of the relationships between changes in MNREAD or CGS and changes in CMT
  We evaluated two types of changes in MNREAD values, CGS, and CMT: the amount of change (value posttreatment—value pretreatment) and percentage change ([value posttreatment—value pretreatment]/value pretreatment). Univariate regression analyses between each visual function parameter and CMT were performed using Wald tests.
4. Estimation of the pretreatment CMT thresholds at which the most significant changes in improvement in the visual function parameters occurred
  Two types of changes in visual function parameters were evaluated: the amount of change and percentage change. We identified the thresholds of CMT at which the most significant changes in improvement in visual function parameters (i.e., the changes associated with the lowest *p-*values) were obtained. We divided the patients into 2 groups (n vs N-n; 1≤ n≤N-1) based on the pretreatment CMT for each visual parameter and determined the CMT at which the lowest *p-*value was obtained for each parameter. The Mann-Whitney test was used for statistical analysis.
5. Evaluation of the effects of the site of BRVO (superior or inferior) on visual function parameters
  The subjects were divided into two groups: subjects with BRVO involving the superior lesion of the macula and those with BRVO involving the inferior lesion of the macula.

The visual function parameters (BCFVA, BCNVA, RA, MRS, CPS, CS, and GS) and CMT in the involved eyes before treatment were evaluated according to the involved site by a linear regression model. The visual function parameters, CMT, and binocular MNREAD parameters before treatment were set as the response variables. The involved site was set as an explanatory variable. The corresponding parameters for the fellow eyes were also included as explanatory variables to control for individual variation.

The amounts of change or percentage changes after treatment of all of the visual function parameters and CMT in the treated eyes were set as the response variables. The involved site was set as the explanatory variable. The corresponding parameters for the affected eyes before treatment were included as explanatory variables to correct for the baseline levels. The amount of change or percentage change after treatment in one of the binocular MNREAD parameters was set as the response variable in each test. The involved site was set as the explanatory variable. The corresponding parameter for the fellow eye was also included as an explanatory variable to control for individual variation.

All comparisons were performed using Wald tests.

## Results

Characteristics and descriptive statistics of the measured variables are presented in S1 Table.

### Visual function parameters and CMT after treatment

**Fig 1:**
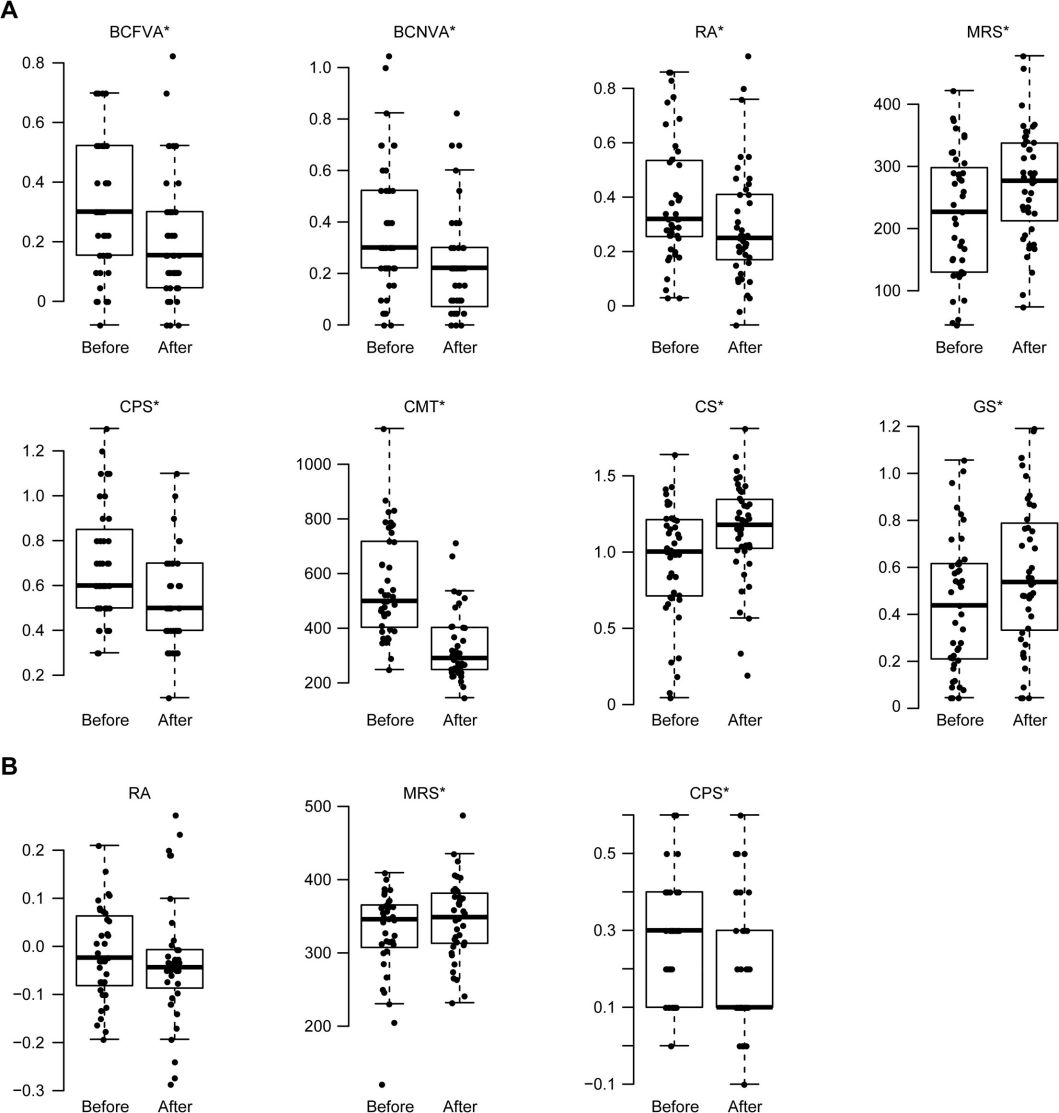
Evaluation of improvements in visual function parameters after ranibizumab treatment of BRVO patients. A: Visual parameters in the treated eye. B: Binocular visual parameters. In the box-and-whisker plots, the bottom of each box indicates the first quartile, and the top of the box indicates the third quartile. The band inside the box represents the median. To highlight suspected outliers, the upper whisker is set as the maximum or the third quartile +1.5×IQR, and the lower whisker indicates the minimum or the first quartile -1.5×IQR. The beeswarm plot is a one-dimensional scatter plot with non-overlapping points. Note that because the placement of the dots in figures is randomly determined in the beeswarm plot, the dots are sometimes merged. * Significant improvement at *p* < 0.05.

The values of the visual function parameters and CMT in the treated eyes before and after treatment are shown in Fig 1. The improvements after treatment are presented in S2 Table. All visual function parameters and CMT in the treated eyes improved after treatment. Among the binocular MNREAD parameters, CPS significantly improved, whereas RA and MRS did not.

### The relationships between BCVA and other visual function parameters in the treated eyes

**Fig 2:**
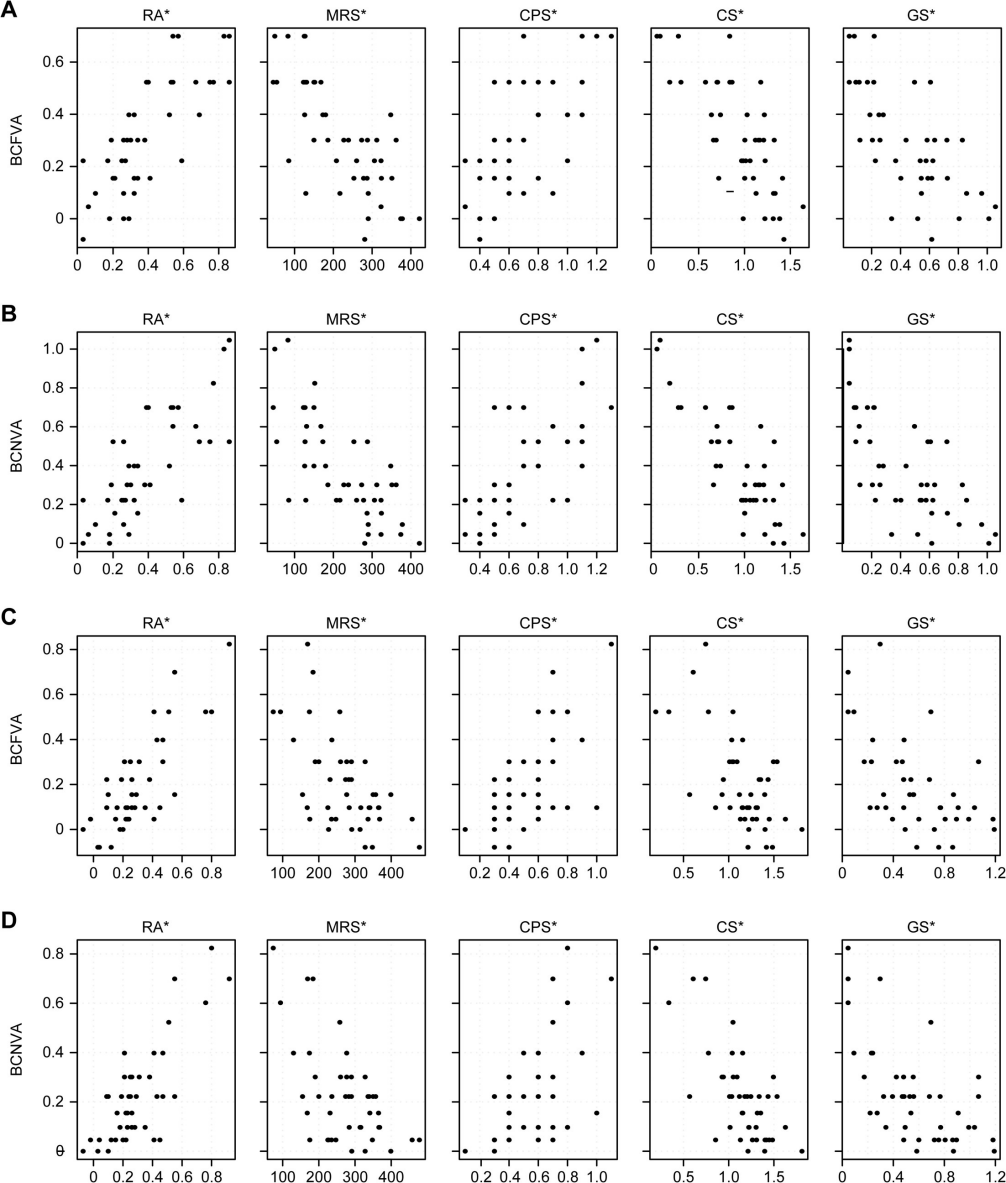
Relationships between BCVA and other visual function parameters in the treated eye. A: Pretreatment BCFVA of the treated eye. B: Pretreatment BCNVA of the treated eye. C: Posttreatment BCFVA of the treated eye. D: Posttreatment BCNVA of the treated eye. * Significant improvement at *p* < 0.01 (0.05/5 = 0.01, Bonferroni correction).

The relationships between BCVA and the other visual function parameters are shown in Fig 2 and S3 Table. As 5 items of visual function were evaluated, a difference was considered significant at *p* < 0.05/5 = 0.01 to account for the multiple comparisons by the Bonferroni method [25,26]. BCFVA and BCNVA were significantly correlated with RA, MRS, CPS, CS, and GS in the treated eyes both before and after treatment.

### The relationships between changes in MNREAD or CGS and changes in CMT

**Fig 3:**
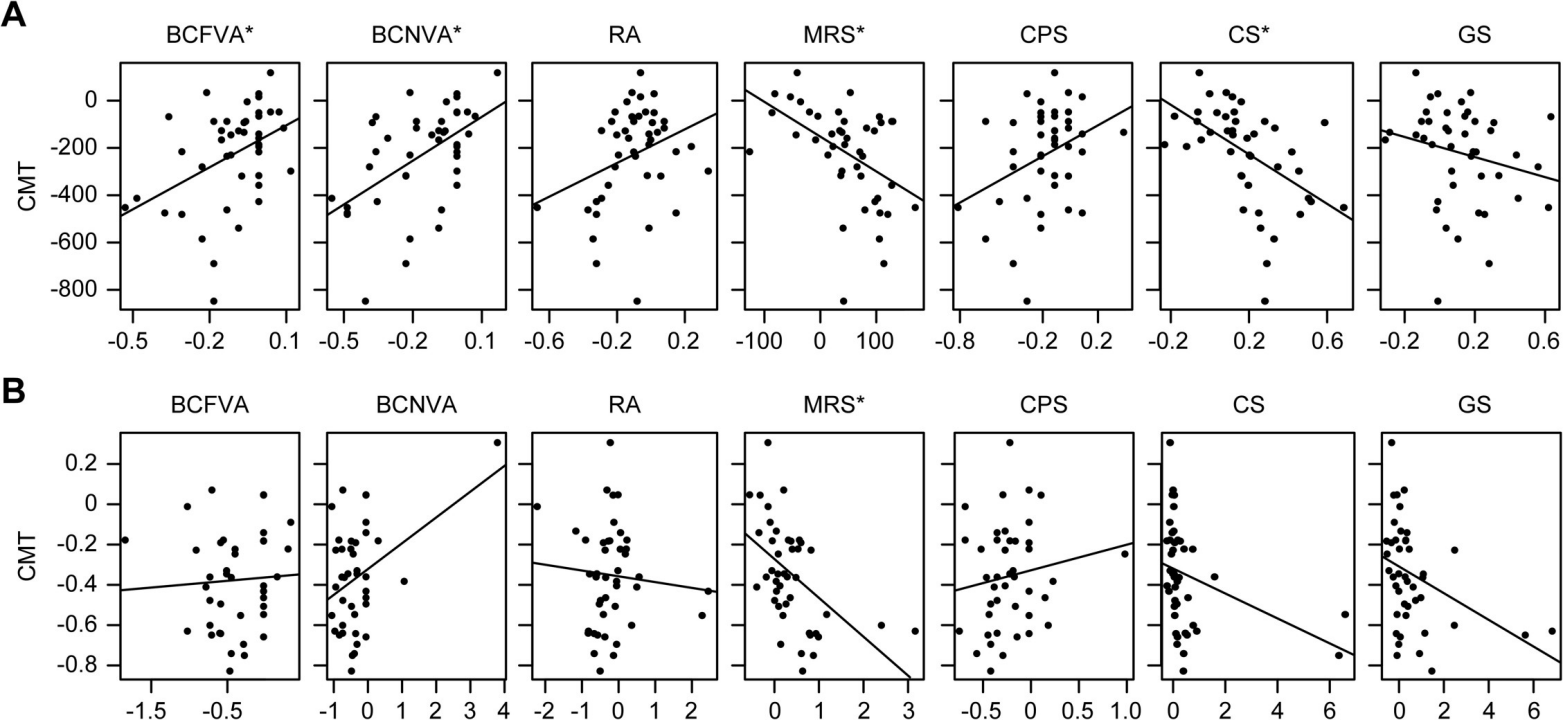
Relationships between changes in CMT and those in other visual function parameters in treated eyes. A: Amount of change. B: Percentage change. * Significant improvement at *p* < 0.007 (0.05/7 = 0.007, Bonferroni correction).

A relationship was considered significant at *p* < 0.05/7 = 0.007 [25,26]. Only the amounts of change in BCFVA, BCNVA, MRS, and CS were significantly correlated with the amount of change in CMT (*p* < 0.007; r = 0.415, 0.528, -0.465, and -0.508, respectively). Furthermore, only the percentage change in posttreatment MRS was significantly correlated with that in CMT (*p* < 0.007; r = -0.511) (Fig 3). The evaluation results are presented in S4 Table.

### CMT thresholds at which the most significant changes in improvement in the visual function parameters were observed

Significant CMT thresholds were obtained for BCVNA, MRS, and CS (S1 Fig, Table 1). Among the amounts of change in these three parameters, the amount of change in MRS yielded the lowest CMT threshold (CMT: 397 μm) in the treated eye (*p* = 0.004929 < 0.005 [0.05/10 = 0.005, Bonferroni correction]). This threshold was also associated with a significantly low *p-*value in terms of the percentage change in MRS in the treated eye (*p* = 0.00001057 < 0.005 [0.05/10 = 0.005, Bonferroni correction]).

**Table 1:** Estimated thresholds for preoperative CMT at which the most significant changes in improvement in the visual function parameters (i.e., the changes associated with the lowest *p*-values) were observed.

No.	Parameter	Threshold for CMT		N	Median (25%, 75%)	*p-*value
1	Treated eye BCFVA (amount of change)	633	≤Threshold	30	-0.048 (-0.121, 0.000)	1.240E-02
			>Threshold	13	-0.176 (-0.301, -0.079)	
2	Treated eye BCNVA (amount of change)	472	≤Threshold	17	0.000 (-0.067, 0.000)	4.425E-04*
			>Threshold	26	-0.204 (-0.351, -0.079)	
3	Treated eye RA (amount of change)	633	≤Threshold	30	-0.050 (-0.122, 0.027)	1.441E-02
			>Threshold	13	-0.280 (-0.310, -0.070)	
4	Treated eye MRS (amount of change)	397	≤Threshold	11	-1.000 (-39.000, 37.000)	4.929E-03*
			>Threshold	32	71.500 (42.000, 109.250)	
5	Treated eye CPS (amount of change)	624	≤Threshold	29	-0.100 (-0.200, 0.000)	7.808E-03
			>Threshold	14	-0.300 (-0.400, -0.200)	
6	Treated eye CS (amount of change)	633	≤Threshold	30	0.126 (0.010, 0.199)	7.497E-06*
			>Threshold	13	0.339 (0.270, 0.473)	
7	Treated eye GS (amount of change)	542	≤Threshold	27	0.087 (-0.047, 0.196)	1.557E-01
			>Threshold	16	0.173 (0.000, 0.306)	
8	Binocular RA (amount of change)	542	≤Threshold	20	-0.000 (-0.045, 0.082)	3.304E-02
			>Threshold	16	-0.057 (-0.125, 0.001)	
9	Binocular MRS (amount of change)	780	≤Threshold	30	3.021 (-15.154, 35.000)	1.131E-01
			>Threshold	6	34.722 (30.838, 53.306)	
10	Binocular CPS (amount of change)	775	≤Threshold	29	-0.100 (-0.200, 0.000)	2.037E-01
			>Threshold	7	0.000 (-0.100, 0.100)	

BCFVA: best-corrected far visual acuity; BCNVA: best-corrected near visual acuity; RA: reading acuity; MRS: maximum reading speed; CPS: critical print size; CS: contrast sensitivity; GS: glare sensitivity.

* indicates significance at *p*<0.005.

### Effects of the site of BRVO on visual function parameters

There were no significant correlations between any of the pretreatment visual function parameters and BRVO site (superior vs. inferior). The pretreatment GS of the affected eye and the CPS of pretreatment binocular vision tended to be worse for superior BRVO-affected eyes than for inferior BRVO-affected eyes (0.05 < *p* = 0.08 < 0.1) (Table 2). No significant relationships between the amounts of change or percentage changes in visual function parameters and BRVO site were observed. A trend of association between the posttreatment CMT of the affected eye and BRVO site was observed (0.05 < *p* = 0.07 < 0.1); when BRVO occurred at a superior site, the CMT of the affected eyes seemed less likely to improve than when it occurred at an inferior site.

**Table 2:** Effects of BRVO site on visual function parameters and CMT.

(A) Vision, pretreatment			
Response variable	Estimate	95% confidence interval	*p-*value
Treated eye BCFVA	0.044	(-0.107, 0.195)	5.61E-01
Treated eye BCNVA	0.062	(-0.123, 0.248)	5.02E-01
Treated eye RA	0.017	(-0.142, 0.176)	8.32E-01
Treated eye MRS	-5.261	(-77.538, 67.015)	8.84E-01
Treated eye CPS	0.046	(-0.14, 0.231)	6.21E-01
Treated eye CMT	-6.344	(-142.104, 129.417)	9.25E-01
Treated eye CS	-0.154	(-0.417, 0.109)	2.43E-01
Treated eye GS	-0.171	(-0.367, 0.024)	8.45E-02
Binocular RA^1^	-0.003	(-0.078, 0.072)	9.37E-01
Binocular MRS^1^	-7.19	(-34.273, 19.894)	5.92E-01
Binocular CPS^1^	0.072	(-0.01, 0.154)	8.13E-02
(B) Vision (amount of change)			
Response variable	Estimate	95% confidence interval	*p-*value
Treated eye BCFVA^2^	-0.045	(-0.142, 0.052)	3.56E-01
Treated eye BCNVA^2^	-0.053	(-0.15, 0.045)	2.80E-01
Treated eye RA^2^	-0.046	(-0.161, 0.069)	4.24E-01
Treated eye MRS^2^	-4.7	(-46.238, 36.838)	8.20E-01
Treated eye CPS^2^	-0.03	(-0.154, 0.094)	6.25E-01
Treated eye CMT^2^	61.86	(-24.362, 148.083)	1.55E-01
Treated eye CS^2^	0.024	(-0.104, 0.153)	7.02E-01
Treated eye GS^2^	-0.047	(-0.203, 0.11)	5.50E-01
Binocular RA^1^	0.033	(-0.049, 0.116)	4.20E-01
Binocular MRS^1^	11.811	(-18.831, 42.453)	4.38E-01
Binocular CPS^1^	-0.002	(-0.132, 0.128)	9.72E-01
(C) Vision (percentage change)			
Response variable	Estimate	95% confidence interval	*p-*value
Treated eye BCFVA^2^	-0.238	(-0.551, 0.075)	1.32E-01
Treated eye BCNVA^2^	-0.156	(-0.713, 0.402)	5.75E-01
Treated eye RA^2^	-0.332	(-0.848, 0.184)	2.01E-01
Treated eye MRS^2^	0.121	(-0.236, 0.478)	4.97E-01
Treated eye CPS^2^	-0.035	(-0.236, 0.166)	7.26E-01
Treated eye CMT^2^	0.128	(-0.013, 0.27)	7.47E-02
Treated eye CS^2^	0.136	(-0.634, 0.905)	7.23E-01
Treated eye GS^2^	-0.059	(-1.042, 0.925)	9.05E-01
Binocular RA^1^	-1.865	(-6.833, 3.103)	4.50E-01
Binocular MRS^1^	0.018	(-0.115, 0.152)	7.81E-01
Binocular CPS^1^	0.306	(-0.243, 0.856)	2.65E-01

BCFVA: best-corrected far visual acuity; BCNVA: best-corrected near visual acuity; RA: reading acuity; MRS: maximum reading speed; CPS: critical print size; CMT: central macular thickness; CS: contrast sensitivity; GS: glare sensitivity.

^1^The coefficient, 95% confidence interval, and *p-*value resulting from the Wald test of the bleeding site were estimated while controlling for the pretreatment values of the treated and fellow eyes.

^2^The coefficient, 95% confidence interval, and *p-*value resulting from the Wald test of the bleeding site were estimated while controlling for the pretreatment value of the treated eye.

## Discussion

We evaluated the effects of an initial single treatment with ranibizumab (Lucentis®) on reading ability, CGS, and CMT in BRVO patients and identified the relationships among visual function parameters. All of the parameters significantly improved after treatment. In the treated eyes, all MNREAD parameters and CGS were significantly correlated with BCFVA and BCNVA both before and after treatment. These results demonstrate that MNREAD and CGS might be reliably used as parameters for visual evaluation. Among the parameters assessed posttreatment, only BCFVA, BCNVA, MRS, and CS exhibited amounts of change that were significantly correlated with the amount of change in CMT (*p* < 0.007), and only MRS exhibited a percentage change that was significantly correlated with that of CMT. Evaluation of the thresholds of CMT revealed that among the visual function parameters, MRS had the lowest threshold CMT (397 μm), and the associated *p-*values for the amount of change and percentage change were both significant. Because this threshold reflects when improvements are likely to occur, these results suggest that MRS can sensitively reflect the dynamics of macular morphology. The present results suggest that MRS and CS can serve as indices of changes in CMT and that MRS is a more sensitive indicator than CS for assessing the effectiveness of anti-VEGF treatment; hence, MRS can be used to make treatment decisions in a PRN regimen.

In this study, BCFVA remained unchanged in 12 patients. Of these patients, 7 exhibited improvement in RA, 8 exhibited improvement in MRS, and 6 exhibited improvement in CPS. These results indicate that reading ability might be more sensitive than visual acuity in indicating the success of BRVO treatment.

No significant relationship was observed between BRVO site and any pretreatment visual function parameter or the amount of change or percentage change in any visual function parameter. However, when BRVO occurred at a superior site, the pretreatment GS of the affected eye and the pretreatment CPS of binocular vision tended to be worse than when BRVO occurred at an inferior site (0.05 < both *p* = 0.08 < 0.1). CPS might be influenced to a greater extent by superior than inferior sites because it is responsible for the lower visual field while reading.

A trend of association between the posttreatment CMT of the affected eye and BRVO site was also found (0.05 < *p* = 0.07 < 0.1). The CMT of the affected eye seemed less likely to improve when BRVO occurred at a superior site than when it occurred at an inferior site. BRVO at a superior site might lead to long-lasting macular edema due to the gravity effect, which in turn might limit CMT improvement.

This study has several limitations. One limitation is that we did not consider subretinal hemorrhage, which might affect the prognosis of visual acuity [27]. Another limitation is that we did not investigate whether the target eye was dominant; this factor might have affected the results. It was not feasible for us to determine which eye was dominant after the occurrence of BRVO. An additional limitation is the limited number of subjects. The main purpose of this study was to investigate the potential utility of MNREAD parameters and CGS measures in evaluating the efficacy of treatment of naive BRVO patients; therefore, we investigated the effects of a single dose of initial anti-VEGF treatment with ranibizumab. However, in most cases, BRVO patients receive repeated ranibizumab injections [1]; therefore, the effects of treatment over a long follow-up period should to be investigated in future research. In addition, we did not consider the type of vein occlusion according to region, i.e., major vessel occlusion vs. macular branch vessel occlusion. This issue should be addressed in future research.

In conclusion, the results of this study highlight the clinical importance of MNREAD parameters and CGS assessment in evaluating the visual function of BRVO patients. Our research suggests that MRS and CS are useful indices and that MRS might be potent therapeutic index for evaluating the success of ranibizumab treatment of BRVO and guiding treatment decisions.

## Supporting information

S1 Fig Estimated thresholds for preoperative CMT at which the most significant changes in improvement in the visual function parameters (i.e., the changes associated with the lowest *p*-values) were observed. (TIF)

S1 Table The analyzed variables. (DOCX)

S2 Table Improvements in visual function parameters and CMT after treatment. (DOCX)

S3 Table Relationships between BCVA and other visual function parameters. (DOCX)

S4 Table Correlations of Changes in CMT with those in the Visual Function Parameters of the Treated Eye (A: Amount of Change, B: Percentage Change).
(DOCX)

S1 Data set (XLSX)
