# Supplementary material for: Potential roles of MNREAD acuity charts and contrast/glare sensitivity in Ranibizumab treatment of branch retinal vein occlusion
Source: PLoS One. 2020 Jul 10;15(7):e0235897. doi: 10.1371/journal.pone.0235897 (PMC7351188; doi:10.1371/journal.pone.0235897)
Supplement: S1 Table — (DOCX) [file pone.0235897.s002.docx]

S1 Table. The Analyzed Variables.

| A. Categorical variables |  |  |  |  |
| --- | --- | --- | --- | --- |
| Serial number | Variable | Level | N |  |
| 1 | Sex | F/M | 21/22 |  |
| 2 | Treated eye | L/R | 14/29 |  |
| 3 | BRVO site | Upper/Lower | 11/32 |  |
| B. Continuous variables |  |  |  |  |
| Serial number | Variable | N (defects excluded) | Mean ± SD | Median (25%, 75%） |
| 1 | Age | 43 | 69.256 ± 8.350 | 71.000 (63.500, 74.500) |
| 2 | Treated eye BCFVA, preoperative | 43 | 0.305 ± 0.213 | 0.301 (0.155, 0.523) |
| 3 | Treated eye BCFVA, postoperative | 43 | 0.202 ± 0.206 | 0.155 (0.046, 0.301) |
| 4 | Treated eye BCNVA, preoperative | 43 | 0.379 ± 0.261 | 0.301 (0.222, 0.523) |
| 5 | Treated eye BCNVA, postoperative | 43 | 0.226 ± 0.199 | 0.222 (0.071, 0.301) |
| 6 | Treated eye RA, preoperative | 43 | 0.382 ± 0.223 | 0.320 (0.255, 0.535) |
| 7 | Treated eye RA, postoperative | 43 | 0.292 ± 0.210 | 0.250 (0.170, 0.410) |
| 8 | Treated eye MRS, preoperative | 43 | 222.721 ± 101.196 | 227.000 (130.000, 298.000) |
| 9 | Treated eye MRS, postoperative | 43 | 271.837 ± 89.704 | 277.000 (212.500, 337.500) |
| 10 | Treated eye CPS, preoperative | 43 | 0.698 ± 0.260 | 0.600 (0.500, 0.850) |
| 11 | Treated eye CPS, postoperative | 43 | 0.530 ± 0.211 | 0.500 (0.400, 0.700) |
| 12 | Fellow eye BCFVA, preoperative | 42 | -0.028 ± 0.108 | -0.079 (-0.079, 0.000) |
| 13 | Fellow eye BCFVA, postoperative | 42 | -0.027 ± 0.115 | -0.079 (-0.079, -0.079) |
| 14 | Fellow eye BCNVA, preoperative | 42 | 0.049 ± 0.096 | 0.000 (0.000, 0.046) |
| 15 | Fellow eye BCNVA, postoperative | 40 | 0.038 ± 0.078 | 0.000 (0.000, 0.011) |
| 16 | Fellow eye RA, preoperative | 42 | -0.035 ± 0.242 | -0.030 (-0.068, 0.055) |
| 17 | Fellow eye RA, postoperative | 39 | -0.015 ± 0.156 | -0.030 (-0.075, 0.010) |
| 18 | Fellow eye MRS, preoperative | 42 | 323.286 ± 65.134 | 336.000 (305.250, 365.500) |
| 19 | Fellow eye MRS, postoperative | 39 | 337.513 ± 57.850 | 352.000 (307.500, 375.000) |
| 20 | Fellow eye CPS, preoperative | 42 | 0.274 ± 0.184 | 0.300 (0.100, 0.400) |
| 21 | Fellow eye CPS, postoperative | 39 | 0.254 ± 0.197 | 0.200 (0.100, 0.300) |
| 22 | Binocular RA, preoperative | 36 | -0.015 ± 0.097 | -0.023 (-0.077, 0.060) |
| 23 | Binocular RA, postoperative | 39 | -0.033 ± 0.126 | -0.043 (-0.087, -0.007) |
| 24 | Binocular MRS, preoperative | 36 | 327.500 ± 60.049 | 345.995 (309.835, 364.861) |
| 25 | Binocular MRS, postoperative | 39 | 346.344 ± 54.554 | 348.870 (313.130, 381.583) |
| 26 | Binocular CPS, preoperative | 36 | 0.272 ± 0.150 | 0.300 (0.100, 0.400) |
| 27 | Binocular CPS, postoperative | 39 | 0.197 ± 0.166 | 0.100 (0.100, 0.300) |
| 28 | Treated eye CMT, preoperative | 43 | 555.279 ± 190.052 | 500.000 (403.500, 718.000) |
| 29 | Treated eye CMT, postoperative | 43 | 330.767 ± 124.052 | 291.000 (249.000, 403.000) |
| 30 | CS, preoperative | 43 | 0.948 ± 0.374 | 1.004 (0.713, 1.213) |
| 31 | CS, postoperative | 43 | 1.144 ± 0.323 | 1.179 (1.025, 1.345) |
| 32 | GS, preoperative | 43 | 0.440 ± 0.284 | 0.438 (0.210, 0.616) |
| 33 | GS, postoperative | 43 | 0.574 ± 0.317 | 0.538 (0.333, 0.788) |
| 34 | Treated eye BCFVA (amount of change) | 43 | -0.103 ± 0.146 | -0.067 (-0.176, 0.000) |
| 35 | Treated eye BCNVA (amount of change) | 43 | -0.153 ± 0.178 | -0.079 (-0.261, 0.000) |
| 36 | Treated eye RA (amount of change) | 43 | -0.090 ± 0.182 | -0.080 (-0.200, 0.015) |
| 37 | Treated eye MRS (amount of change) | 43 | 49.116 ± 65.855 | 47.000 (20.500, 103.500) |
| 38 | Treated eye CPS (amount of change) | 43 | -0.167 ± 0.219 | -0.100 (-0.250, 0.000) |
| 39 | Fellow eye BCFVA (amount of change) | 42 | 0.000 ± 0.055 | 0.000 (0.000, 0.000) |
| 40 | Fellow eye BCNVA (amount of change) | 40 | -0.007 ± 0.049 | 0.000 (-0.011, 0.000) |
| 41 | Fellow eye RA (amount of change) | 39 | 0.028 ± 0.247 | -0.020 (-0.070, 0.050) |
| 42 | Fellow eye MRS (amount of change) | 39 | 14.436 ± 37.897 | 12.000 (-12.000, 46.000) |
| 43 | Fellow eye CPS (amount of change) | 39 | -0.021 ± 0.173 | 0.000 (-0.100, 0.100) |
| 44 | Binocular RA (amount of change) | 36 | -0.020 ± 0.104 | -0.020 (-0.061, 0.036) |
| 45 | Binocular MRS (amount of change) | 36 | 11.812 ± 42.063 | 17.019 (-15.136, 37.116) |
| 46 | Binocular CPS (amount of change) | 36 | -0.075 ± 0.168 | -0.100 (-0.125, 0.000) |
| 47 | Treated eye CMT (amount of change) | 43 | -224.512 ± 208.288 | -163.000 (-335.500, -85.000) |
| 48 | CS (amount of change) | 43 | 0.195 ± 0.202 | 0.173 (0.093, 0.297) |
| 49 | GS (amount of change) | 43 | 0.134 ± 0.214 | 0.114 (-0.004, 0.240) |
| 50 | Treated eye BCFVA (percentage change) | 39 | NaN ± NaN | -0.399 (-0.652, 0.000) |
| 51 | Treated eye BCNVA (percentage change) | 41 | Inf ± NaN | -0.386 (-0.683, 0.000) |
| 52 | Treated eye RA (percentage change) | 43 | -0.145 ± 0.745 | -0.226 (-0.495, 0.037) |
| 53 | Treated eye MRS (percentage change) | 43 | 0.423 ± 0.674 | 0.265 (0.066, 0.644) |
| 54 | Treated eye CPS (percentage change) | 43 | -0.203 ± 0.296 | -0.250 (-0.382, 0.000) |
| 55 | Fellow eye BCFVA (percentage change) | 36 | NaN ± NaN | -0.000 (-0.000, -0.000) |
| 56 | Fellow eye BCNVA (percentage change) | 15 | Inf ± NaN | -0.263 (-1.000, 0.538) |
| 57 | Fellow eye RA (percentage change) | 39 | 0.094 ± 2.319 | -0.000 (-1.000, 0.721) |
| 58 | Fellow eye MRS (percentage change) | 39 | 0.067 ± 0.160 | 0.039 (-0.035, 0.154) |
| 59 | Fellow eye CPS (percentage change) | 38 | Inf ± NaN | 0.000 (-0.333, 0.175) |
| 60 | Binocular RA (percentage change) | 36 | -1.405 ± 6.490 | -0.191 (-1.075, 0.537) |
| 61 | Binocular MRS (percentage change) | 36 | 0.062 ± 0.195 | 0.048 (-0.043, 0.128) |
| 62 | Binocular CPS (percentage change) | 35 | Inf ± NaN | -0.333 (-0.688, 0.000) |
| 63 | Treated eye CMT (percentage change) | 43 | -0.353 ± 0.254 | -0.358 (-0.546, -0.179) |
| 64 | CS (percentage change) | 43 | 0.554 ± 1.384 | 0.150 (0.074, 0.478) |
| 65 | GS (percentage change) | 43 | 0.674 ± 1.436 | 0.320 (-0.018, 0.771) |

BCFVA: best-corrected far visual acuity; BCNVA: best-corrected near visual acuity; RA: reading acuity; MRS: maximum reading speed; CPS: critical print size; CMT: central macular thickness; CS: contrast sensitivity; GS: glare sensitivity.
