# Supplementary material for: Potential roles of MNREAD acuity charts and contrast/glare sensitivity in Ranibizumab treatment of branch retinal vein occlusion
Source: PLoS One. 2020 Jul 10;15(7):e0235897. doi: 10.1371/journal.pone.0235897 (PMC7351188; doi:10.1371/journal.pone.0235897)
Supplement: S2 Table — (DOCX) [file pone.0235897.s003.docx]

**S2 Table. Improvements in Visual Function Parameters and CMT after Treatment.**

| Eye | Visual function |  | Pretreatment | Posttreatment | Wilcoxon test *p-*value |
| --- | --- | --- | --- | --- | --- |
| Treated eye | BCFVA | N | 43 | 43 | 5.387E-05* |
|  |  | Mean (SD) | 0.305 (0.213) | 0.202 (0.206) |  |
|  |  | Min.-Max. | -0.079–0.699 | -0.079–0.824 |  |
|  |  | Median (25%, 75%) | 0.301 (0.155, 0.523) | 0.155 (0.046, 0.301) |  |
| Treated eye | BCNVA | N | 43 | 43 | 6.936E-06* |
|  |  | Mean (SD) | 0.379 (0.261) | 0.226 (0.199) |  |
|  |  | Min.-Max. | 0.000–1.046 | 0.000–0.824 |  |
|  |  | Median (25%, 75%) | 0.301 (0.222, 0.523) | 0.222 (0.071, 0.301) |  |
| Treated eye | RA | N | 43 | 43 | 2.036E-03* |
|  |  | Mean (SD) | 0.382 (0.223) | 0.292 (0.210) |  |
|  |  | Min.-Max. | 0.030–0.860 | -0.070–0.920 |  |
|  |  | Median (25%, 75%) | 0.320 (0.255, 0.535) | 0.250 (0.170, 0.410) |  |
| Treated eye | MRS | N | 43 | 43 | 7.101E-05* |
|  |  | Mean (SD) | 222.721 (101.196) | 271.837 (89.704) |  |
|  |  | Min.-Max. | 45.000–422.000 | 74.000–478.000 |  |
|  |  | Median (25%, 75%) | 227.000 (130.000, 298.000) | 277.000 (212.500, 337.500) |  |
| Treated eye | CPS | N | 43 | 43 | 1.075E-05* |
|  |  | Mean (SD) | 0.698 (0.260) | 0.530 (0.211) |  |
|  |  | Min.-Max. | 0.300–1.300 | 0.100–1.100 |  |
|  |  | Median (25%, 75%) | 0.600 (0.500, 0.850) | 0.500 (0.400, 0.700) |  |
| Treated eye | CMT | N | 43 | 43 | 6.099E-08* |
|  |  | Mean (SD) | 555.279 (190.052) | 330.767 (124.052) |  |
|  |  | Min.-Max. | 249.000–1131.000 | 146.000–713.000 |  |
|  |  | Median (25%, 75%) | 500.000 (403.500, 718.000) | 291.000 (249.000, 403.000) |  |
| Treated eye | CS | N | 43 | 43 | 1.109E-07* |
|  |  | Mean (SD) | 0.948 (0.374) | 1.144 (0.323) |  |
|  |  | Min.-Max. | 0.045–1.640 | 0.193–1.811 |  |
|  |  | Median (25%, 75%) | 1.004 (0.713, 1.213) | 1.179 (1.025, 1.345) |  |
| Treated eye | GS | N | 43 | 43 | 3.237E-04* |
|  |  | Mean (SD) | 0.440 (0.284) | 0.574 (0.317) |  |
|  |  | Min.-Max. | 0.045–1.057 | 0.045–1.191 |  |
|  |  | Median (25%, 75%) | 0.438 (0.210, 0.616) | 0.538 (0.333, 0.788) |  |
| Binocular | RA | N | 36 | 39 | 2.550E-01 |
|  |  | Mean (SD) | -0.015 (0.097) | -0.033 (0.126) |  |
|  |  | Min.-Max. | -0.193–0.210 | -0.287–0.273 |  |
|  |  | Median (25%, 75%) | -0.023 (-0.077, 0.060) | -0.043 (-0.087, -0.007) |  |
| Binocular | MRS | N | 36 | 39 | 1.307E-01 |
|  |  | Mean (SD) | 327.500 (60.049) | 346.344 (54.554) |  |
|  |  | Min.-Max. | 120.858–409.575 | 232.100–488.319 |  |
|  |  | Median (25%, 75%) | 345.995 (309.835, 364.861) | 348.870 (313.130, 381.583) |  |
| Binocular | CPS | N | 36 | 39 | 1.687E-02* |
|  |  | Mean (SD) | 0.272 (0.150) | 0.197 (0.166) |  |
|  |  | Min.-Max. | 0.000–0.600 | -0.100–0.600 |  |
|  |  | Median (25%, 75%) | 0.300 (0.100, 0.400) | 0.100 (0.100, 0.300) |  |

BCFVA: best-corrected far visual acuity; BCNVA: best-corrected near visual acuity; RA: reading acuity; MRS: maximum reading speed; CPS: critical print size; CMT: central macular thickness; CS: contrast sensitivity; GS: glare sensitivity. * indicates significance at *p*<0.05.
