# Supplementary material for: Potential roles of MNREAD acuity charts and contrast/glare sensitivity in Ranibizumab treatment of branch retinal vein occlusion
Source: PLoS One. 2020 Jul 10;15(7):e0235897. doi: 10.1371/journal.pone.0235897 (PMC7351188; doi:10.1371/journal.pone.0235897)
Supplement: S3 Table — (DOCX) [file pone.0235897.s004.docx]

S3 Table. Relationships between BCVA and Other Visual Function Parameters.

| Condition of comparison eye | Time | Vision visual acuity | MNREAD-J vision | Reliability evaluation correlation coefficient | *p-*value |
| --- | --- | --- | --- | --- | --- |
| Treated eye | Pretreatment | BCFVA | RA | 0.781 | 6.537E-10* |
| Treated eye | Pretreatment | BCFVA | MRS | -0.738 | 1.636E-08* |
| Treated eye | Pretreatment | BCFVA | CPS | 0.684 | 4.339E-07* |
| Treated eye | Pretreatment | BCFVA | CS | -0.756 | 4.644E-09* |
| Treated eye | Pretreatment | BCFVA | GS | -0.716 | 6.716E-08* |
| Treated eye | Pretreatment | BCNVA | RA | 0.779 | 7.903E-10* |
| Treated eye | Pretreatment | BCNVA | MRS | -0.730 | 2.789E-08* |
| Treated eye | Pretreatment | BCNVA | CPS | 0.700 | 1.762E-07* |
| Treated eye | Pretreatment | BCNVA | CS | -0.816 | 2.615E-11* |
| Treated eye | Pretreatment | BCNVA | GS | -0.690 | 3.084E-07* |
| Treated eye | Posttreatment | BCFVA | RA | 0.779 | 7.473E-10* |
| Treated eye | Posttreatment | BCFVA | MRS | -0.602 | 1.962E-05* |
| Treated eye | Posttreatment | BCFVA | CPS | 0.635 | 4.729E-06* |
| Treated eye | Posttreatment | BCFVA | CS | -0.626 | 7.121E-06* |
| Treated eye | Posttreatment | BCFVA | GS | -0.544 | 1.609E-04* |
| Treated eye | Posttreatment | BCNVA | RA | 0.774 | 1.120E-09* |
| Treated eye | Posttreatment | BCNVA | MRS | -0.593 | 2.835E-05* |
| Treated eye | Posttreatment | BCNVA | CPS | 0.590 | 3.157E-05* |
| Treated eye | Posttreatment | BCNVA | CS | -0.735 | 2.045E-08* |
| Treated eye | Posttreatment | BCNVA | GS | -0.628 | 6.582E-06* |

BCFVA: best-corrected far visual acuity; BCNVA: best-corrected near visual acuity; RA: reading acuity; MRS: maximum reading speed; CPS: critical print size; CS: contrast sensitivity; GS: glare sensitivity. * Indicates significance at *P*<0.01.
