# Supplementary material for: Potential roles of MNREAD acuity charts and contrast/glare sensitivity in Ranibizumab treatment of branch retinal vein occlusion
Source: PLoS One. 2020 Jul 10;15(7):e0235897. doi: 10.1371/journal.pone.0235897 (PMC7351188; doi:10.1371/journal.pone.0235897)
Supplement: S4 Table — Correlations of Changes in CMT with those in the Visual Function Parameters of the Treated Eye (A: Amount of Change, B: Percentage Change). (DOCX) [file pone.0235897.s005.docx]

S4 Table. Correlations of Changes in CMT with Those in the Visual Function Parameters of the Treated Eye (A: Amount of Change, B: Percentage Change).

| A. Amount of change |  |  |  |  |
| --- | --- | --- | --- | --- |
| Explanatory variable | Efficacy evaluation |  |  |  |
| Visual function parameter | Regression coefficient | 95% Confidence interval | *p-*value | Correlation coefficient |
| BCFVA (best-corrected far visual acuity) | 591.767 | (182.51, 1001.023) | 5.663E-03* | 0.415 |
| BCNVA (best-corrected near visual acuity) | 617.638 | (304.03, 931.247) | 2.768E-04* | 0.528 |
| RA (reading acuity) | 355.871 | (13.327, 698.414) | 4.210E-02 | 0.311 |
| MRS (maximum reading speed) | -1.470 | (-2.353, -0.587) | 1.686E-03* | -0.465 |
| CPS (critical print size) | 326.653 | (44.933, 608.373) | 2.414E-02 | 0.343 |
| CS | -523.272 | (-802.87, -243.674) | 5.011E-04* | -0.508 |
| GS | -212.217 | (-511.371, 86.937) | 1.595E-01 | -0.218 |
| B. Percent change |  |  |  |  |
| BCFVA | 0.033 | (-0.155, 0.222) | 7.207E-01 | 0.059 |
| BCNVA | 0.128 | (0.029, 0.227) | 1.235E-02 | 0.387 |
| RA | -0.029 | (-0.136, 0.078) | 5.911E-01 | -0.084 |
| MRS | -0.193 | (-0.295, -0.091) | 4.610E-04* | -0.511 |
| CPS | 0.127 | (-0.141, 0.395) | 3.441E-01 | 0.148 |
| CS | -0.062 | (-0.116, -0.007) | 2.724E-02 | -0.337 |
| GS | -0.066 | (-0.118, -0.014) | 1.350E-02 | -0.374 |

BCFVA: best-corrected far visual acuity; BCNVA: best-corrected near visual acuity; RA: reading acuity; MRS: maximum reading speed; CPS: critical print size; CS: contrast sensitivity; GS: glare sensitivity. * Indicates significance at *p*<0.007.
